# Dynamics of Wind Setdown at Suez and the Eastern Nile Delta

**DOI:** 10.1371/journal.pone.0012481

**Published:** 2010-08-30

**Authors:** Carl Drews, Weiqing Han

**Affiliations:** 1 Department of Atmospheric and Oceanic Sciences, University of Colorado at Boulder, Boulder, Colorado, United States of America; 2 NCAR Earth System Laboratory, National Center for Atmospheric Research, Boulder, Colorado, United States of America; Universidade de Vigo, Spain

## Abstract

**Background:**

Wind setdown is the drop in water level caused by wind stress acting on the surface of a body of water for an extended period of time. As the wind blows, water recedes from the upwind shore and exposes terrain that was formerly underwater. Previous researchers have suggested wind setdown as a possible hydrodynamic explanation for Moses crossing the Red Sea, as described in Exodus 14.

**Methodology/Principal Findings:**

This study analyzes the hydrodynamic mechanism proposed by earlier studies, focusing on the time needed to reach a steady-state solution. In addition, the authors investigate a site in the eastern Nile delta, where the ancient Pelusiac branch of the Nile once flowed into a coastal lagoon then known as the Lake of Tanis. We conduct a satellite and modeling survey to analyze this location, using geological evidence of the ancient bathymetry and a historical description of a strong wind event in 1882. A suite of model experiments are performed to demonstrate a new hydrodynamic mechanism that can cause an angular body of water to divide under wind stress, and to test the behavior of our study location and reconstructed topography.

**Conclusions/Significance:**

Under a uniform 28 m/s easterly wind forcing in the reconstructed model basin, the ocean model produces an area of exposed mud flats where the river mouth opens into the lake. This land bridge is 3–4 km long and 5 km wide, and it remains open for 4 hours. Model results indicate that navigation in shallow-water harbors can be significantly curtailed by wind setdown when strong winds blow offshore.

## Introduction

Wind setdown occurs in shallow coastal areas when strong winds blow offshore. When wind stress acts for several hours on a body of water, the free water surface acquires a low-angle tilt. This tilt causes the water on the upwind side to recede from the original shoreline, leaving exposed mud flats on the bottom. Wind setdown is opposite to storm surge and comparable in vertical displacement, although wind setdown is less well known because it usually poses no danger to lives and property. Wind setdown events on the order of 2 m were recorded by measuring stations at the western end of Lake Erie on December 1–2, 2006, and January 30–31, 2008 [1]. Lake Erie is the southernmost of the Great Lakes between Canada and the United States. Cedar Key Harbor in Florida, USA, experienced a 1.0 m drop in water level on September 6, 2004 as Hurricane Frances passed through, then rose to 1.5 m above sea level 9 hours later [1].

### 1.1 Research Background

**Figure 1:**
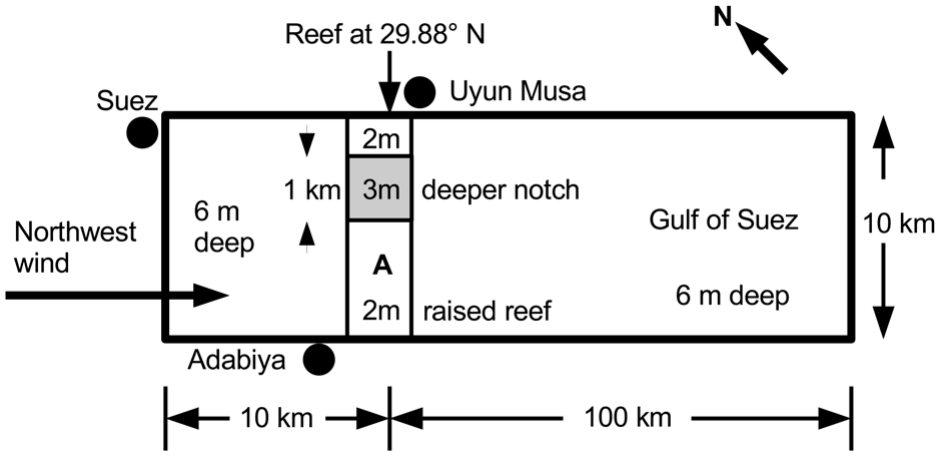
Map of the idealized Gulf of Suez. The reef at 29.88° North latitude is not of uniform depth today, but contains several deeper “notches” that were present before the Suez Canal was built [40]. Here we use a single notch to demonstrate how variations in the reef depth may delay the crossing. Point A on the raised portion of the reef is used to compare with the notch (see Figures 5 and 6). The wind blows from the Northwest, as studied by Nof and Paldor [3] and Voltzinger and Androsov [4].

Tidal ebbs can cause water to recede from the original shoreline and expose the bottom of the sea, and it is easy to imagine wind stress causing similar behavior. Previous researchers analyzed a more interesting phenomenon: the formation of a **land bridge** extending from one original shoreline to another, with water remaining on both sides of the bridge. They suggested that a large number of people (the Israelites) could possibly “cross the sea” on such a dry passage. Nof & Paldor [2], [3] found analytical solutions to the differential equations of a 1-dimensional (1-D) model that govern the water's free surface under wind stress forcing. They used a wind blowing from the Northwest, aligned with the primary axis of the Gulf of Suez. They suggested that the water receded and an underwater reef emerged from the sea when the wind setdown occurred. The proposed reef is at 29.88° N; it extends about 10 km under the Gulf of Suez from a point 3 km Southeast of Adabiya across to Uyun Musa on the eastern shore (see Figure 1 for an idealized map of Suez).

Voltzinger & Androsov [4] used a 3-dimensional (3-D) model to simulate the reef at 29.88° N in the Gulf of Suez. The modern reef at approximately 10 m deep was raised to a uniform depth of 3 m below sea level. They suggested that wind blowing initially from the Northeast gradually changed direction to blow from the Northwest, becoming aligned with the axis of the Gulf. Voltzinger & Androsov calculated an exposure time of 4 hours for the reef, using a wind speed that ramps up from 0 m/s and blows at 33 m/s for 12 hours. They noted that the dividing ridge between the Suez basin and the rest of the Gulf of Suez significantly increases the time to reach a steady-state solution.

Since the isthmus of Suez is a low-lying area that in ancient times contained several shallow bodies of water and interconnecting river channels, there are additional sites that are interesting from a hydrodynamic perspective. It is important to investigate these other possibilities. We consider a site at the northern end of the isthmus that exhibits similar interaction between wind and water.

### 1.2 Study Location

Scientific literature from the 19^th^ century contains a description of a wind setdown event that occurred in the eastern Nile delta. Major-General Alexander B. Tulloch of the British Army reported this event happening on Lake Manzala in January or February 1882:

One day, when so employed [surveying] between Port Said and Kantarah, a gale of wind from the eastward set in and became so strong that I had to cease work. Next morning on going out I found that Lake Menzaleh, which is situated on the west side of the [Suez] Canal, had totally disappeared, the effect of the high wind on the shallow water having actually driven it away beyond the horizon, and the natives were walking about on the mud where the day before the fishing-boats, now aground, had been floating. When noticing this extraordinary dynamical effect of wind on shallow water, it suddenly flashed across my mind that I was witnessing a similar event to what had taken place between three and four thousand years ago, at the time of the passage of the so-called Red Sea by the Israelites.

…

**Mr. M. Rooke:** I should like to ask the present depth of Lake Menzahleh [sic] near Port Said?

**Tulloch:** It is only about 5 feet or 6 feet.

**Rooke:** Where was the water driven to?

**Tulloch:** It was “packed up” to the north-west.

**Rooke:** Could you see it in any way?

**Tulloch:** It was seven miles off. It had absolutely disappeared.

[5]

In this paper we retain the easterly wind direction described by Tulloch, noting that the primary axis of Lake Manzala is oriented East-West. Under this easterly wind forcing we investigate the possible separation of a body of water, with the presence of water remaining on both sides of a dry passage. This choice of direction restricts the hydrodynamic possibilities to those bodies of water near Sinai that have a long East-West extent. The Gulf of Suez, which was the study area in Nof & Paldor [2] and Voltzinger & Androsov [4], is oriented primarily North-South. Consequently, an easterly wind there will not produce a dry region with water remaining on both sides.

**Figure 2:**
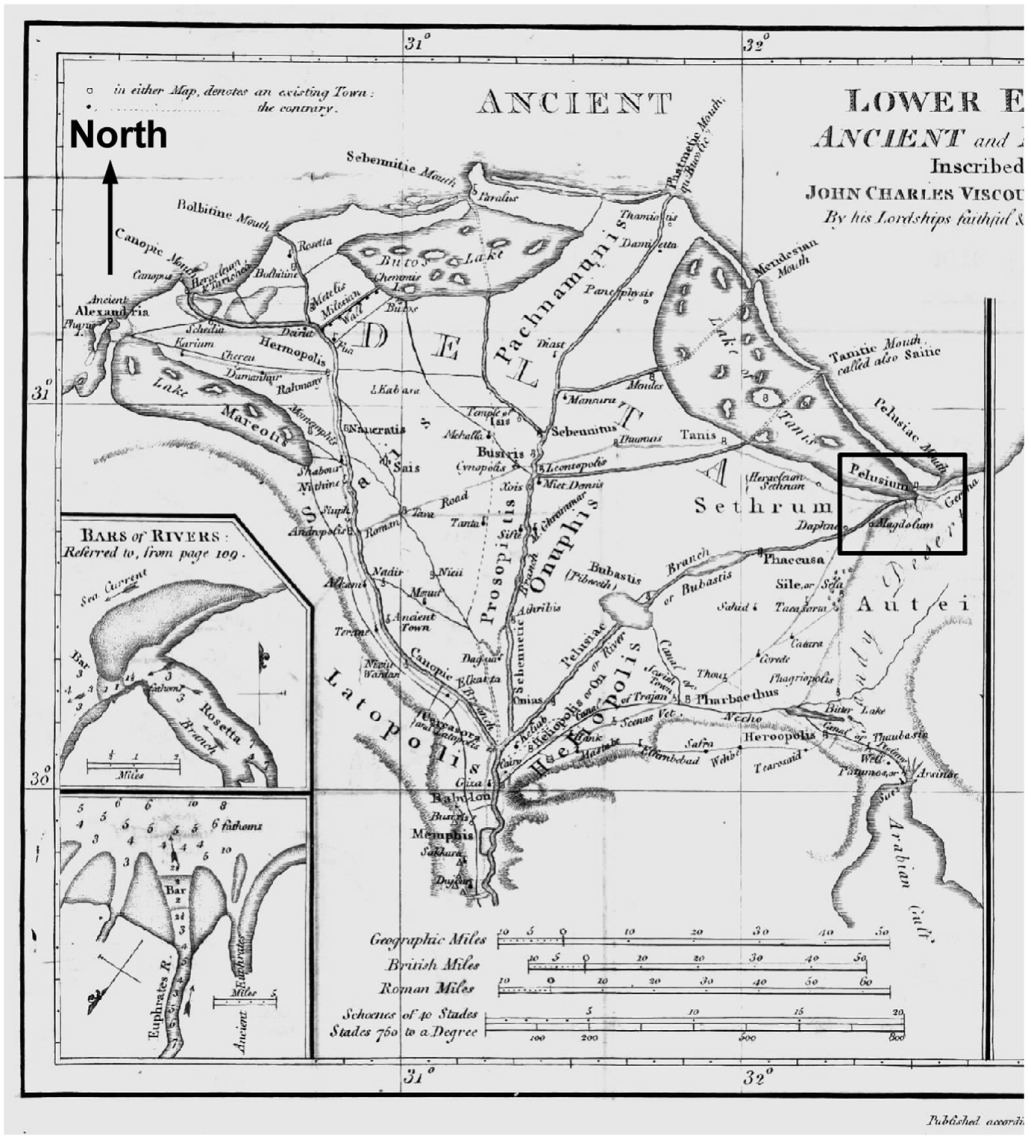
James Rennell's reconstruction of the Nile delta according to Herodotus. Our study location is on the right side of the figure (solid rectangle: 30.8°–31.2° North, 32.2°–32.6° East), from Sethrum to a point between Pelusium and Magdolum, where the Lake of Tanis and the Pelusiac branch of the Nile come together. The solid rectangle also shows the outline of Figure 3.

We set our study during Egypt's New Kingdom period, with a nominal date of 1250 BC. The exact date is not crucial to our study. This era contains considerable historical interest, and provides the opportunity to use archaeological and geological studies of the “Ways of Horus” East of the Suez Canal [6]. During the Late Bronze Age, the Nile river in Egypt had seven mouths opening onto the Mediterranean Sea [7]. Based on the descriptions of Herodotus, James Rennell published a map of the Nile Delta region [8] during the Greek classical period (shown here in Figure 2). He drew the coastal lagoon then known as the Lake of Tanis extending into the eastern Nile delta to Pelusium. An angular bend in the ancient bodies of water occurred at the eastern edge of the Nile delta, where the Pelusiac branch of the Nile river flowed into the Lake of Tanis (solid rectangle on the right side of Figure 2, between Pelusium and Magdolum). The Lake of Tanis and the Pelusiac branch had a long extent in the East-West direction (>25 km); during wind setdown this extent of shallow water can produce a vertical displacement of 2 m. Archaeologist James Hoffmeier and geologist Stephen Moshier have created a map of the Ways of Horus that shows the confluence of the Pelusiac branch and the Lake of Tanis in greater detail during the New Kingdom period (Hoffmeier's Figure 5 [9]), based upon extensive field work. Their Figure 5 shows a paleolagoon opening northward to the open sea at Kedua.

**Figure 3:**
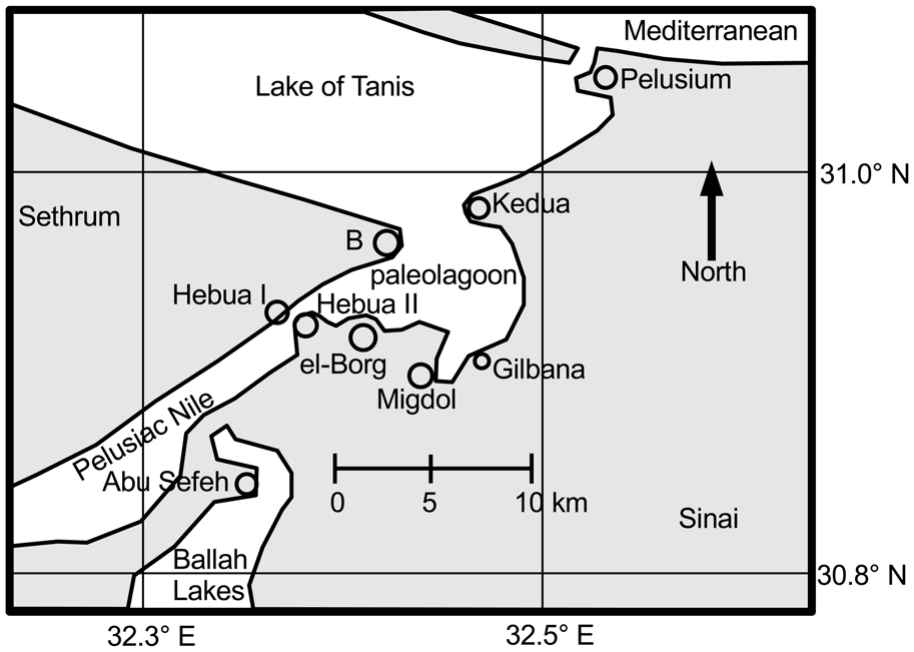
Reconstruction of the geography Southwest of Pelusium. This map is outlined as a solid rectangle in Figure 2. We analyze the gap where the paleolagoon opens to the North at Kedua. This diagram draws from the following sources: Hoffmeier's Figure 5 [9], Rennell [8], Sneh and Weissbrod [13], Moshier & El-Kalani [6], Google Earth [23], SRTM3 data [22], and Kitchen [41] Figure 28.

Based on the above data, we analyzed a possible division of water at this Kedua Gap. We suggest that the area labeled “Open Sea” in Hoffmeier's Figure 5 [9] was instead the Lake of Tanis, and this brackish lagoon was separated from the Mediterranean Sea by a line of sandy barrier islands. Our alternate configuration (Figure 3) reflects that the Nile river forms a wave-dominated delta with smoothly curving arcuate coastlines [10](p.302). These arcs and coastal lagoons are maintained by sediment plumes from the Nile mouths, swept eastward by the alongshore current [11]. The currents and sediment would naturally form a coastal sandbar, dividing the shallow lagoon from the deeper open sea. We plan to investigate this issue further using the sediment cores described by Stanley et al. [12]. An ocean general circulation model (OGCM) with easterly wind forcing and topography that resembles that of 1250 BC is used to simulate a wind setdown event.

Satellite images, the SRTM3 terrain data, and the Israeli Geological Survey [13] reveal an unusually straight line running Southwest from Pelusium. This feature is the Pelusium Line, a tectonic feature [14]. The Pelusium Line shows up on the ground as a sandstone ridge upon which Hebua I is situated [9](p. 42). The ridge intersects the Suez Canal 4 km North of Qantara. The Pelusium Line provides a few valuable points of geographical stability among the shifting sands of the Nile delta [6]. Being somewhat resistant to erosion, we used it to help locate the ancient course of the Pelusiac branch and the associated coastal lagoons. Table 1 provides coordinates for locating these sites.

**Table 1:** Coordinates for locating and mapping the important sites for this research.

Name	Alternate name	Latitude (decimal degrees North)	Longitude (decimal degrees East)	Notes
Bubastis	Tell Basta	30.5722	31.5124	Southeast of modern Zagazig.
Damietta		31.4204	31.8075	
Daphnae	Tahpanhes	30.8606	32.1714	Inflow from the Pelusiac branch.
Gilbana		30.9307	32.4736	Smaller land bridge.
Hebua I	Tjaru	30.9352	32.3669	New Kingdom fort.
Hebua II		30.9319	32.3811	
Kedua	Tell Qedua	30.9833	32.4755	Endpoint of the land bridge.
Kedua Gap		30.9812	32.4553	Center of the land bridge.
Migdol	T-78	30.9059	32.4415	Magdolum?
paleolagoon		30.945	32.45	Visible on satellite photographs.
Pelusium	Tell el-Farama	31.0424	32.5400	
Point B		30.9666	32.4234	Starting point for the Kedua crossing.
Tell Abu Sefeh	Abu Seifa	30.8606	32.3543	Promontory at Ballah Lakes.
Tell el-Borg		30.9234	32.4126	Alternate site for Migdol.

## Methods

### 2.1 The Regional Ocean Modeling System (ROMS)

The ROMS is a modern OGCM that can be configured to any ocean region ranging from local to basin scale. ROMS implements the primitive equations with a free sea surface and a terrain-following s-coordinate in the vertical dimension. Values of s are between -1 and 0, which represent the fraction of the local water column thickness measured from the surface. The model numerics are described in Moore et al. [15] and Shchepetkin & McWilliams [16]. ROMS has been used to simulate the circulation in a variety of different ocean regions [17], [18], including shallow coastal estuaries [19]. The wind-driven components have been compared favorably with observations of storm surge [20]. ROMS implements a scheme for wetting and drying whereby the water's edge can advance to cover formerly dry land, or recede and expose the underlying bathymetry. This scheme employs a critical depth, which is the minimum water depth that the ocean model will resolve. We set the critical depth to 0.10 m; ROMS treats any surface covered to this depth or less as “dry land”. The land-sea mask is set to 30 m above sea level, beyond any possible storm surge.

### 2.2 Model Basin and Topography Modification

**Figure 4:**
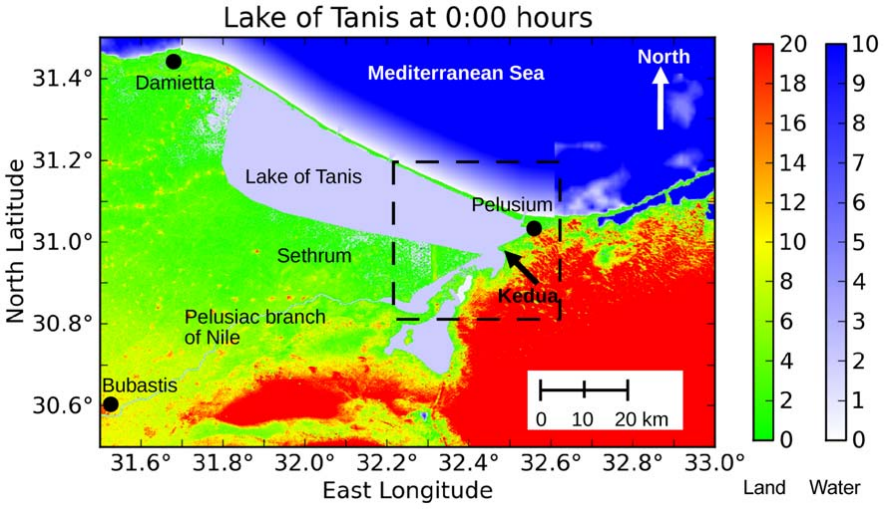
Geography during the Egyptian New Kingdom after terrain modifications. Green represents land surface, and blue-white represents shallow lagoons. The modern Lake Manzala has been extended eastward to reach Pelusium and form the ancient Lake of Tanis. The Pelusiac branch of the Nile has also been restored. The dashed rectangle shows the outline of Figure 8. Bathymetry is given in section 2.2. Color scales are in meters.

The ROMS is configured to the eastern Nile delta (31.5°E–33°E, 30.5°N–31.5°N) with 86 m horizontal grid resolution: 1800 grid points from West to East, and 1200 grid points from South to North. Figure 4 shows this region. The model is run in 2-D barotropic mode for the Lake Tanis case study (section 3.2) and in 3-D for the reef test case (section 3.1). The 3-D mode uses Mellor-Yamada level 2.5 turbulence closure for vertical mixing [21]. The Mellor-Yamada mixing algorithm helps to calculate the response of shallow water to wind stress. The mean water density is 1025 kg/m^3^. The domain boundaries are no-slip. The simulation time step is 1 second, and state variables are recorded every 6 minutes.

Scientific sources do not agree on the exact geography of the eastern Nile delta during the Late Bronze Age. Stanley and Warne have shown that this area was indeed coastal (not completely dry) between four and two thousand years ago [11], [14], [6]. Although any historical reconstruction of this period must necessarily involve uncertainty, our geographical configuration is within the range of published scientific literature. Hoffmeier and Moshier's geography [9] represents the best combination of archaeological and geological field work; our overall approach simply adds a coastal sandbar separating the Lake of Tanis from the open sea [11].

The United States Geological Survey (USGS) provides worldwide topographic data on their web site from the Shuttle Radar Topography Mission (SRTM), at a grid resolution of 3 arc-seconds, or 86 m at 31° North latitude [22]. The bathymetry is supplied from Smith & Sandwell data at a horizontal grid resolution of 30 arc-seconds [22]. The authors merged the high-resolution topography with the lower-resolution bathymetry by linear interpolation. We then created a ROMS domain of the eastern Nile delta by modifying the SRTM terrain to reflect the geography of the Late Bronze Age. These terrain modifications were guided by Figures 2 and 3, satellite photographs from Google Earth [23] and NASA World Wind [24], and geological surveys of the northeastern Nile delta [13], [6]. We followed the general principle that low-lying areas were formerly underwater, and that ancient lake beds are often revealed by modern vegetation patterns. Specific modifications to the SRTM3 terrain are listed below.

- Moved the Mediterranean coastline southward by about 10 km, forming a straight line of coastal barrier dunes between Damietta and Pelusium [11]. This operation reverses the rapid strand-plain accretion described by Goodfriend & Stanley [25]. Figure 4 shows the revised coastline.
- Created the Lake of Tanis between Damietta and Pelusium [11]. The modern Lake Manzala has an average depth of 1.3 m [26]. Herodotus indicates that there were navigable channels from Tanis and Pelusium [27], so we have set the Lake of Tanis to be 2 m deep.
- Opened the Pelusiac, Tanitic, and Mendesian mouths of the Nile [7]. These mouths are channels from Lake of Tanis through the coastal strip to the Mediterranean Sea.
- Created two connected lagoons West and East of Hebua. The western lagoon is described by Sneh et al. [28] and Bietak [29] . The eastern lagoon is described by Hoffmeier [9] and Moshier & El-Kalani [6]. The lagoons are 2 m deep.
- Created the Ballah Lakes using Manfred Bietak's map of East Delta and North Sinai (Figure 1 of Bietak [29]; Google Earth; SRTM terrain data). This lake is also 2 m deep, except for the marsh in the Northeast which is 0.5 m deep.
- Restored the Pelusiac branch of the Nile from Bubastis to Daphnae. The depth of this river channel is set to 3 m [30]. We set our study in the season of spring, when the Nile river within the delta was at its lowest level [31](p. 452), [32]. The course of the Pelusiac is guided by satellite imagery, modern canals, and geophysical surveys [33].

The raised banks of the Suez Canal were left in place. Figure 4 shows the modified geography. All lagoons and lakes are 2.0 m deep. The water surface is initially at sea level except for the Pelusiac branch West (upstream) of Daphnae. Supporting File S1 contains the terrain modifications in KML format suitable for viewing with Google Earth.

The customary way to model river inflow in ROMS is to add a point source of water flux at sea level, usually at a domain boundary. This method will not work here because the inflow must adjust to the wind stress. Instead, we have modeled the river inflow at Daphnae as a long channel that rises gradually up to Bubastis, and have placed the point source there. The net inflow at Daphnae is the combined result of gravitational flow opposed by the wind stress. The height of the free water surface at Bubastis is set initially to 4.8 m. This value represents a linear interpolation between Daphnae and the present water surface height at Cairo of 9 m [22].

The volume inflow at Bubastis is set to 150 m^3^/s, which is estimated from the Damietta branch hydraulics measured by Moussa and Aziz [30]. They state: “The cross section hydraulics characteristics: average velocity V = 0.2 to 0.5 m/s, average depth D = 2 to 4.5 m and top width T = 200 to 250 m.” From these values we take the depth at the center of the channel to be 3 m during low water in the spring, and assume a trapezoidal cross section spread over 3 grid cells. We use 250 m for the top width because it is close to a distance we can model with the 86 m ROMS grid.
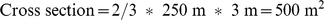
(1)Assume the average flow velocity is 0.3 m/s in spring:
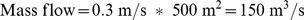
(2)The modeled Pelusiac channel is 260 m wide (3 grid cells) and 3 m deep in the center.

### 2.3 Wind Forcing

We chose the velocity of our modeled wind to be 28 m/s (100 km/h) at 10 m above the surface for the Lake of Tanis case study. This wind speed represents a medium-strength tropical storm on the Saffir-Simpson scale. A 100 km/h wind would be memorable but would not prevent travel on foot. This value is comparable to the previous studies. The surface wind stress *τ* is calculated from the drag coefficient *C_d_*, the air density *ρ*, and the wind velocity *u* at 10 m (denoted u10),
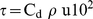
(3)where *C_d_* increases with the wind speed as the water surface becomes more rough. Weisberg & Zheng [34] state that the Large & Pond [35] formulation for the drag coefficient reaches its maximum value at wind velocity 25 m/s and is constant at higher speeds. Thus the drag coefficient increases in linear fashion with the wind velocity until 25 m/s is reached:
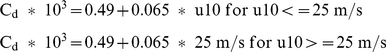
(4)This formulation yields
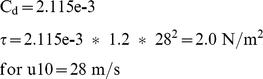
(5)Higher wind speeds, such as 33 m/s applied by Voltzinger & Androsov [4], are also tested (see section 3.1). In comparison, Resio & Westerink [36] use a maximum value for the drag coefficient of *C_d_* = 2.5e-3, then augment *C_d_* with an *R*-factor that represents the transfer of wave momentum. Our value of *C_d_* = 2.115e-3 is within the range used by existing studies.

Recent research indicates that the air-sea drag coefficient *C_d_* levels off and decreases as wind speeds approach hurricane force. The Weisberg and Zheng [34] formulation used in this paper models this effect by cutting off the Large and Pond [35] increase at 25 m/s. Thus, ROMS uses the same value of *C_d_* (2.115e-3) for winds of 28 m/s and 33 m/s. Powell et al. [37] found that *C_d_* increases as surface winds approach 33 m/s, then decreases thereafter. Jarosz et al. [38] found the maximum *C_d_* to occur near 32 m/s. From these studies it is evident that our formulation for *C_d_* in equation (4) should be reduced if used for wind speeds greater than 33 m/s. Jarosz' Figure 3 provided a best-fit parabolic equation for the drag coefficient at wind speeds from 20 m/s to 45 m/s:
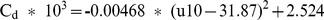
(6)Equation (6) produces a value of *C_d_* = 2.45e-3 for u10 = 28 m/s, and 2.52e-3 for 33 m/s. When compared with the Jarosz formulation, our model underestimates the wind stress for both wind speeds used.

The ROMS is forced by the uniform easterly winds with a speed of 28 m/s, starting from a state of no motion. Our modeled wind blows from the East for 12 hours, the same basic period of time used by Voltzinger and Androsov [4].

## Results

In this section, we first test the underwater reef case proposed by Nof & Paldor [2], and then report our new results for the Lake of Tanis.

### 3.1 Reef Test Case

A series of experiments were performed using the 3-D ROMS for the idealized Suez basin with an underwater reef (Figure 1), as proposed by Nof & Paldor [2]. See Table 2 for descriptions of the experiments; we use the notation RN for the Reef cases, where N is an integer representing the case number. This domain corresponds roughly to the Gulf of Suez, although the Red Sea at Suez today is deeper. For all experiments, the model integration starts from a state of rest with uniform potential temperature, salinity, and thus density under uniform northwesterly wind stress forcing. In experiments Reef case 3 (R3), R4 and R5, the wind stress ramps up from 0 to 2.7 N/m^2^ (equivalent to 33 m/s wind speed) over a period of 12 hours, remains constant until 24 hours, and then drops immediately to zero. We used a ramp function here as the most expedient way to reach the steady-state solutions reported by Nof & Paldor. Voltzinger & Androsov [4] also used a wind speed that steadily ramps up to the maximum value, then acts for 12 hours. Experiments R1 and R2 are the same as R3 and R4, except that wind speed is 28 m/s.

**Table 2:** ROMS experiments conducted in the Suez basin with an underwater reef.

Exp.	Northwesterly forcing wind	Reef condition	Duration of reef exposure:	Notch dry:
R1	28 m/s	2 m deep reef with uniform depth	10 hours	-
R2	28 m/s	2 m deep reef with 3 m deep notch	10 hours	Never
R3	33 m/s	2 m deep reef with uniform depth	12 hours	-
R4	33 m/s	2 m deep reef with 3 m deep notch	15 hours	Never
R5	33 m/s	3 m deep reef with uniform depth	9 hours (only to half-width)	-

Wind is from the Northwest, aligned with the primary axis of the Gulf of Suez. The reef varies in depth from 2 to 3 m. The reef exposure time depends on the wind stress and the reef bathymetry. The lower notch in the reef never becomes exposed.

**Figure 5:**
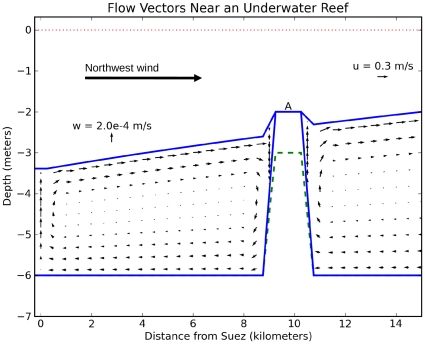
Cross section of currents in the Reef test case R4. The cross section is taken from left to right at Point A in Figure 1. The dotted horizontal red line represents the original sea level (depth = 0). The upper and lower solid blue lines represent the sea surface and sea bottom, respectively. The dashed green line represents the notch. Wind at 33 m/s blows from the left, representing a northwesterly wind as in Nof and Paldor [2]. The water has uniform potential temperature, salinity and thus density. The raised portion of the reef (Point A) emerges above sea level from 10 through 24 hours of our model integration to provide a partially dry crossing. The 3 m deep notch, however, remains below sea level.

Figure 5 shows a vertical cross section of the water level and circulation patterns from experiment R4 near the underwater ridge in the idealized Gulf of Suez, taken from left to right at Point A in Figure 1. This figure compares favorably to the analytical solution of Nof & Paldor's Figure 4 and Voltzinger & Androsov's Figure 7. Under a strong northwesterly wind forcing, the reef is exposed above the sea level.

**Figure 6:**
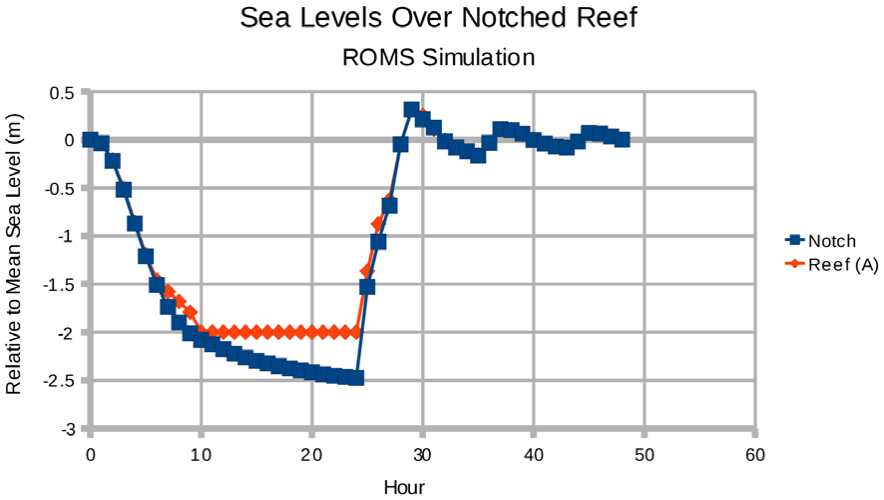
Time series of water levels at the reef. The time series is taken at the raised portion of the reef proper (Point A in Figure 1), and at the deeper “notch” in the reef. In experiment R4 the reef becomes dry at 2 m below sea level at 10 hours, and remains dry until the wind ceases at 24 hours. The water level in the notch drops at nearly the same rate, but does not ever reach its “fully dry” level of 3 m below sea level.

It appears that for the underwater reef mechanism, its timing requires a reef of uniform depth in order to produce a completely dry reef (Table 2, R1, R3 and R5). When a 3 m deep notch is present, the notch never becomes completely dry (R2 and R4). Voltzinger & Androsov's Figures 8 and 9 show rivulets of water about 0.2 m deep still streaming over the ridge, but they do not attach much importance to these. We find that any deeper sections of the reef will form rivers of seawater that will lengthen the basin draining time and block the attempted passage. Initially the draining passage from the upwind basin is the full 10 km wide; when the water level drops below 2 m, the channel becomes only 1 km wide. Thus the draining channel becomes constricted as the water level drops, and this constriction reduces the overall flow rate. Figure 6 shows in a pair of time series that the reef proper at Point A becomes dry from 10 hours through 24 hours, but the 3m-deep notch itself remains below sea level even after 24 hours of model integration. The notch reaches a minimum depth of 0.5 m at 24 hours, with a current of 0.9 m/s. Such a river 1 km wide would pose a formidable barrier to anyone attempting the passage. Both points on the reef are quickly inundated when the wind ceases. Sensitivity tests to wind strength show that the notched reef never dries out completely (Table 2), even when we used the greater wind speeds of 33 m/s from Voltzinger & Androsov [4]. In comparison, a 2 m reef of uniform depth is dry from 13 through 24 hours (R3 of Table 2). A 3 m reef of uniform depth is dry from 16 through 24 hours, but only to 1/2 of its full width (R5).

These model results indicate that the underwater reef scenario for a crossing contains the following drawbacks:

1. Any irregularity in the reef level greatly increases the drying time;
2. The actual reef at 29.88° N is presently too deep to emerge at these wind speeds.

### 3.2 Simulation of Lake of Tanis

The Tanis experiments are performed using the 2-D ROMS barotropic mode. In this paper the abbreviation TN refers to the simulation experiments, where N is an integer representing a single Tanis case (Table 3). For all experiments, the model integration starts from a state of rest with uniform potential temperature, salinity and density (1025 kg/m^3^) as for the reef test case, but under uniform easterly wind stress forcing in T1–T3. The wind stress lasts for 12 hours, and the model runs for 24 hours. In experiment T1, the wind stress of 2.0 N/m^2^ (equivalent to 28 m/s wind speed) exerts force on the ocean surface beginning at time zero, blows for a period of 12 hours, and then relaxes immediately to zero. We used a step function for Lake of Tanis instead of a ramp function because we are interested in the transient behavior and the timing of this event.

**Table 3:** ROMS experiments conducted on the Lake of Tanis.

Experiment	Configuration	Passage dry for:
T1	Wind speed 28 m/s	3.9 hours
T2	Wind speed 33 m/s	7.4 hours
T3	No Coriolis force. 28 m/s.	3.6 hours
T4	No wind	
T5	Larger domain	4.0 hours
T6	28 m/s at 40° N of E	
T7	33 m/s at 40° N of E	
T8	Tanitic levees	3.8 hours
T9	28 m/s at 30° S of E	
T10	28 m/s E, no river inflow	4.1 hours
T11	33 m/s, larger domain	7.4 hours
T12	28 m/s, no coastal sandbar	
T13	33 m/s, no coastal sandbar	4.6 hours
T14	28 m/s, Kedua Gap 3 km	4.0 hours

The duration of the dry passage at Kedua depends on the wind strength, with stronger winds producing a longer exposure time. The Coriolis force extends the crossing time by 18 minutes.

#### 3.2.1 Idealized Bend Case

**Figure 7:**
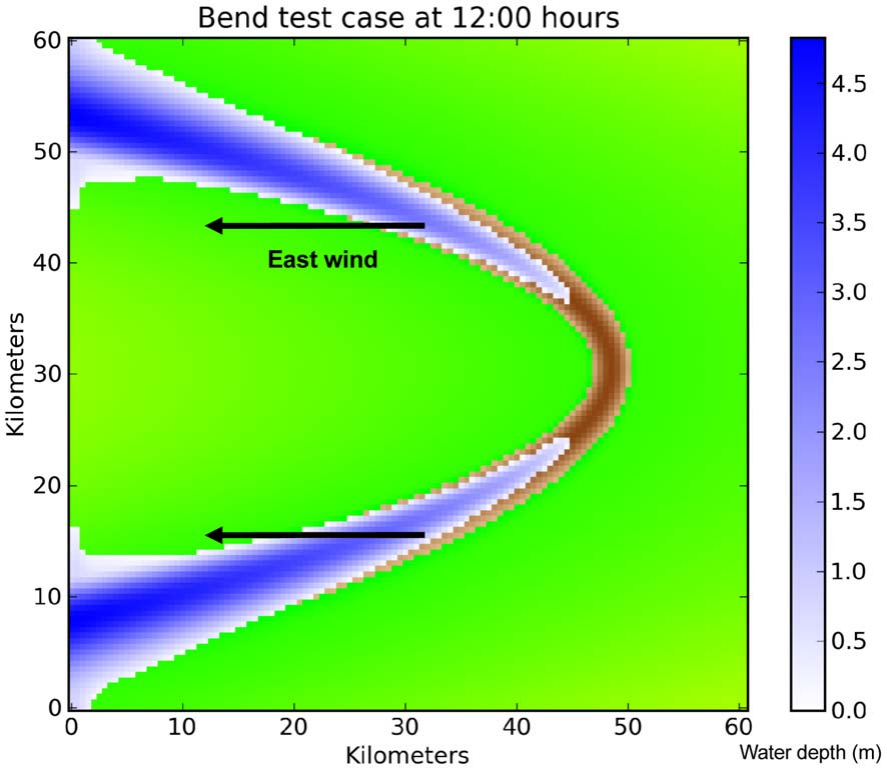
An idealized angular bend of water in the Lake of Tanis. Green represents land surface, white and blue represent water, and brown represents exposed lake bottom. Wind blows from the right at 28 m/s for 12 hours, and wind stress is uniform over the entire domain. The channel is originally 2 m deep and the banks are 1 m high; this bathymetry is comparable to lakes and rivers in the Nile delta during the spring.

To demonstrate the mechanism of water partition for the Lake of Tanis (section 3.2.2), we first discuss the idealized water bend case. As discussed in section 1.2, an angular bend of water occurs in our domain of interest (solid rectangle on the right side of Figure 2), and this angle is important for generating the presence of water on both sides of the land bridge. The angular bend represents a new hydrodynamic mechanism for parting the waters. To test this mechanism, we created an idealized oxbow lake and applied a uniform easterly wind of 28 m/s. As shown in Figure 7, at the eastern head of the bend, mud flats are exposed by the receding water after 12 hours of model integration. These mud flats represent the area of crossing, and the crossing party would observe water to their left and to their right.

#### 3.2.2 Realistic Topography Case

**Figure 8:**
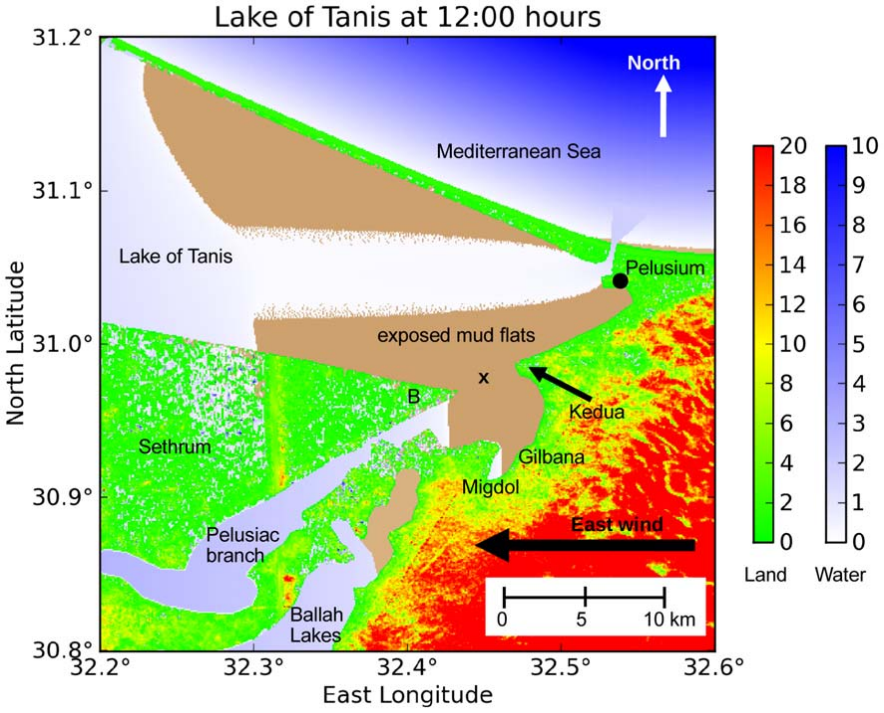
The ROMS solution (experiment T1) after 12 hours, from point B to Kedua. Green represents land surface, white represents shallow lagoons, and brown represents exposed mud flats. Note the Pelusiac jet, a stream of shallow water extending toward the West from the Pelusiac mouth. Only the extreme eastern end of Lake of Tanis (shown here) becomes dry; the central portion remains wet, and the western end overflows the shoreline. The ‘x’ shows the location of Figure 9. Color scales are in meters.

Our modified topography, based on historical data (section 2.2), contains a water bend in our model basin where the Pelusiac Nile flows into the Lake of Tanis. Figure 8 shows the ROMS solution after 12 hours of model integration under 28 m/s easterly wind forcing (T1 of Table 3). The site of interest is between point B at the eastern tip of the Sethrum peninsula, and Kedua, about 4 km to the East. A traversable dry gap in the waters opens here at 9:36 hours, where it appears feasible for a number of people to make their way across the exposed mud flats. The midpoint of the land bridge is at (30.9812° N, 32.4553° E). The passage is about 5 km wide initially, and it later expands up to 6 km wide. This land bridge remains continuously open until 13:30 hours, leaving 3.9 hours for the company to cross the Kedua Gap.

The Pelusiac mouth of the Nile during the Greek classical period was the channel at Pelusium opening onto the Mediterranean Sea (Figure 8). This connection inhibits the wind setdown on the Lake of Tanis, but not enough to prevent a land bridge from forming. The inflow there from the Mediterranean Sea forms a high-velocity stream (−0.8 m/s) running straight downwind across the mud flats, and this Pelusiac jet limits the width of the land bridge to the North. The jet is 0.2 m deep; it remains narrow and does not spread southward. Without the Pelusiac jet, the crossing party would not see water on their left side.

The wind relaxes at 12:00 hours. The Rossby number of the Kedua Gap during the return surge (at 17:00 hours) is given by: R_o_ = V / L *f* = 0.5 m/s / (3.5 km * 7.5e-5 /s) = 1.9, where V is the characteristic velocity, L is the length scale, and *f* is the Coriolis parameter for this latitude. This value indicates that the Coriolis force will have a small effect but nonlinearity will have a large effect on the system. We ran the ROMS model with Coriolis parameter *f* = 0 to quantify the *f* effects (Table 3, experiment T3). Comparing T1 with *f* = 7.5e-5 and T3 with *f* = 0, we find that Ekman transport in T1 makes the land bridge 2 grid cells (170 m) wider on the North side by directing the Pelusiac jet farther North, away from the Kedua Gap. Ekman transport also reduces the sea level by 0.045 m along the Mediterranean coast, thereby enhancing slightly the wind setdown in the Lake of Tanis (figures not shown). The opening and closing times of the land bridge are slightly changed, reducing the passage time by 18 minutes for T3.

**Figure 9:**
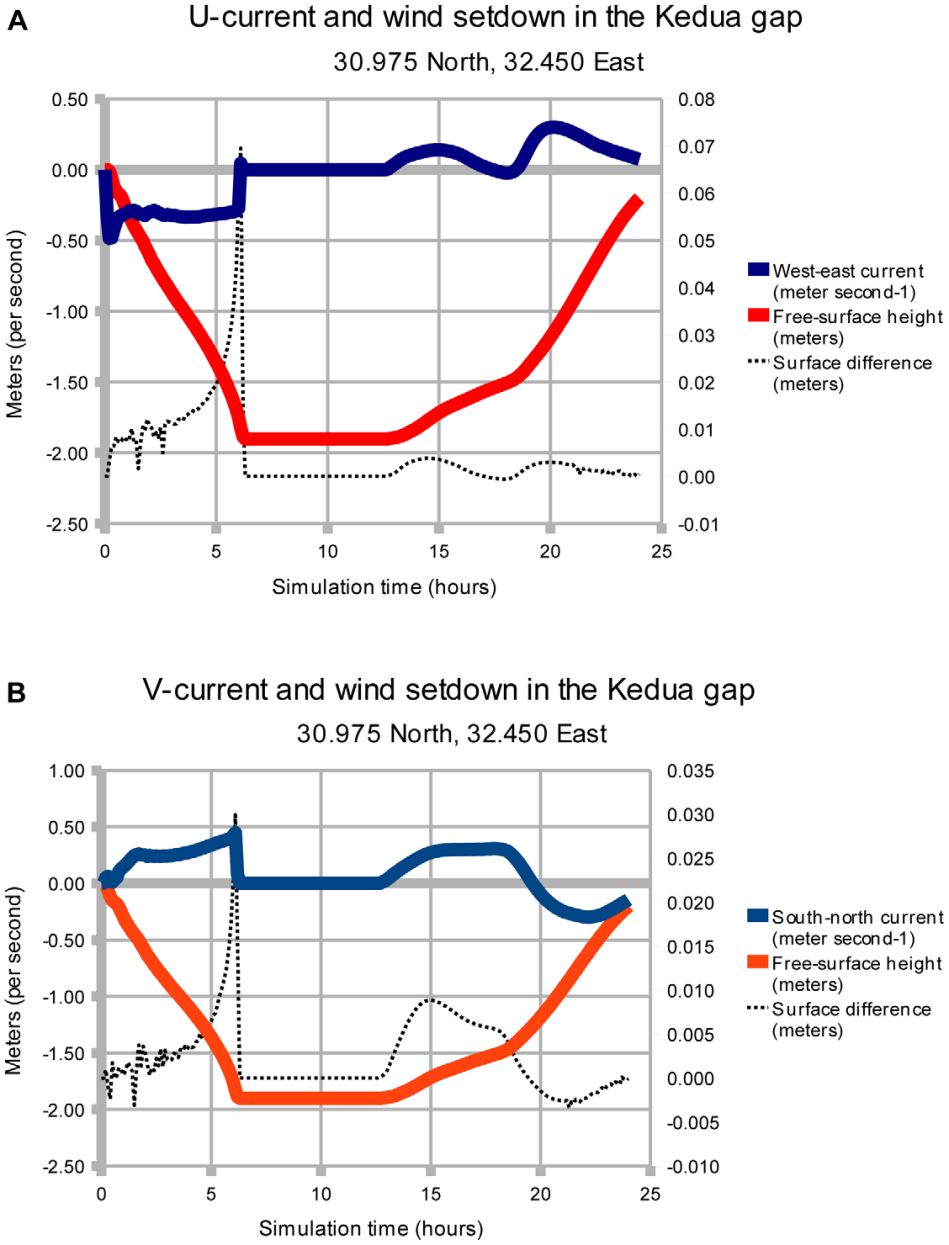
Time series of water level and currents within the Kedua passage. The time series is taken midway between point B and Kedua (at ‘x’ in Figure 8). The upper solid blue line represents the currents, and the lower solid red line represents the water level. The wind stress is constant from 0 to 12 hours, producing dry ground with no current in this particular grid cell after 6 hours. At 12 hours the wind drops to zero and the water surges back into this grid cell 1 hour later. Part (a) represents the West-East u-current, and (b) represents the South-North v-current. The returning wave flows from West to East in (a), and varies in direction in (b). The dotted black line represents the difference in height of the water surface for two grid points in the Kedua Gap, 86 m apart in the current direction; the vertical scale in meters is on the right. The difference in surface height has been calculated as (West - East) and (South - North) so that the plot may be compared visually with the current.

The current within the Kedua Gap exhibits a series of strong North-South flows, as shown in Figure 9(b). At 15:00 hours the meridional current in the Kedua Gap (referred to as v-current here) is strongly positive (toward the North), indicating a return wave from South to North. Since the wind stress is only from the East and the Coriolis effect is small, this v-current must be caused by pressure gradient forces within the gap. The Lake of Tanis is an enclosed coastal region that restricts water movement; under wind stress forcing the free-surface gradient acquires a North-South component in several places. Note that the plots of surface difference (black dotted line) and current in Figure 9 are correlated, except for the first 6 hours of Figure 9(a). The similar shape of the two curves confirms that the currents within the gap are pressure-driven, except for the first 6 hours of 9(a) when wind stress drives the current instead.

**Figure 10:**
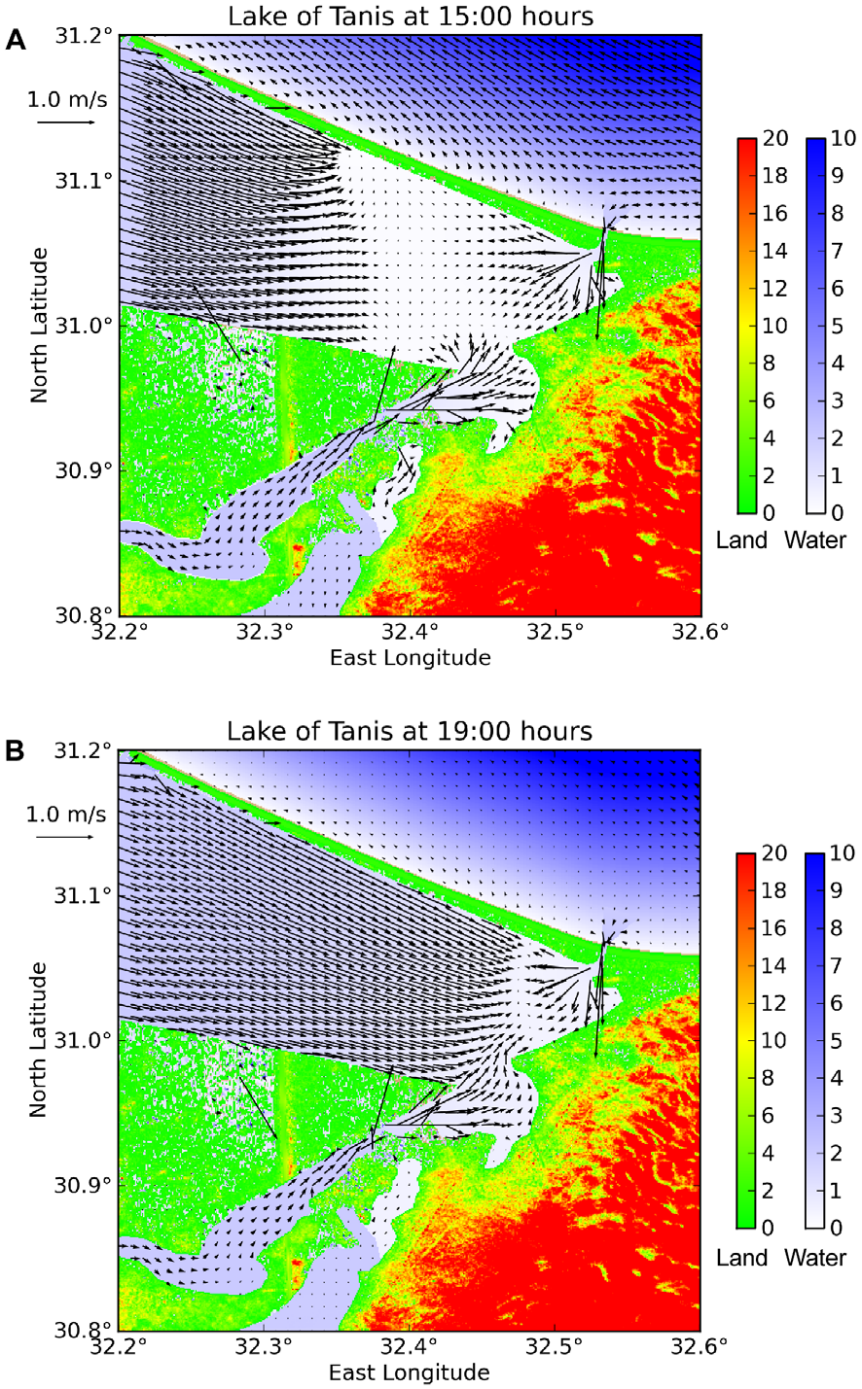
Current vectors at 15:00 and 19:00 hours. The wind ceases at 12:00 hours, and the return wave inundates the exposed mud flats. The initial surge through the Kedua Gap is from South to North (a); the following surge is from West to East (b). For clarity, only 1/10 of the flow vectors are shown in each horizontal dimension. Color scales are in meters. Place names are shown in Figure 8.

Figure 10 shows a map of currents near the crossing site at 3 and 7 hours after the wind ceases. The return wave should travel at the shallow-water speed 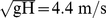; where g = 9.8 m/s^2^ is the acceleration of gravity, and H = 2 m is the lagoon depth. The surge would behave like a hydraulic jump (tidal bore), and would appear as an advancing wall of churning water. This finding suggests that if a crossing actually took place here, any debris field of military artifacts should be found to the North of the gap (Figure 10(a)). The u-current at the gap also increases to +0.3 m/s after the wind ceases. This secondary surge would carry any remaining debris eastward toward the ancient coastline between Kedua and Pelusium (Figure 10(b)).

A second land bridge on a smaller scale occurs at Gilbana, across the southern arm of the eastern paleolagoon (Figure 8). This passage opens at 8:18 hours and closes at 12:48 hours, leaving 4.5 hours for a dry crossing.

#### 3.2.3 Sensitivity to Wind and Asymmetry of the Bend

The duration of the modeled crossing at Kedua is sensitive to the wind speed and topography. If the wind speed is increased to 33 m/s (a Category 1 hurricane) as used by Voltzinger & Androsov [4], the land bridge becomes completely dry at 7:06 hours. This passage becomes wet again at 14:30 hours, leaving 7.4 hours for a group of people to cross (Table 3, experiment T2). The crossing is delayed in all experiments by a narrow (∼200 m) stream of water along the North shore at point B that takes about an hour to disappear completely.

The asymmetry of the angular bend affects the wind setdown situation. Figure 8 clearly shows that the Lake of Tanis arm has receded about 15 km farther West of Kedua than the paleolagoon/Pelusiac arm. This difference may be explained by observing that the Tanis basin provides a much longer East-West extent for the wind stress to act (80 km vs. 30 km for the Pelusiac arm). Wind setdown is proportional to the length of the body of water upon which the wind stress is applied [36].

#### 3.2.4 Pelusiac River Inflow

Table 3 shows the entire suite of ROMS model experiments. Experiment T4 tests the modeled river inflow at Daphnae. Under no wind stress, the Pelusiac channel shows an initial current of 0.6 m/s, which gradually reduces to 0.44 m/s after 24 hours of simulation as the river channel drains eastward. These current values are somewhat greater than the average velocities of 0.2–0.5 m/s reported by Moussa and Aziz [30] for the modern Damietta Branch. Since the modeled channel at 260 m is slightly wider than the top width they reported (200–250 m), it therefore carries more water, and overestimates the inflow at Daphnae. At the modeled grid resolution (86 m) it is not possible to model the river channel more accurately. Under wind stress a minor storm surge travels up the Pelusiac, similar to the surge that traveled up the Mississippi channel during Hurricane Katrina 2005 [39]. When the wind ceases at 12:00 hours, the backed-up water resumes its eastward gravitational flow. T4 shows that our modeled river inflow at Daphnae behaves in a sufficiently realistic manner under wind stress and gravity, over the short duration of this experiment.

Experiment T10 measures the impact of the Daphnae inflow at the Kedua Gap. The point source at Bubastis is set to zero, and the water in the Pelusiac branch of the Nile is eliminated entirely, leaving an empty river channel from Bubastis to Daphnae. In this model configuration the land bridge at Kedua remains open for 4.1 hours (12 minutes longer than the standard configuration T1). Therefore the Daphnae inflow has only a minor impact on the dry passage at Kedua.

#### 3.2.5 Other Experiments

T5 and T11 test the domain extent. The northern boundary of the standard ROMS domain is at 31.5° North latitude, and this limit provides a model grid that is computationally manageable. However, the boundary constricts the ocean flow at Damietta between the coast and the domain wall (Figure 4). Does this unnatural constriction affect the experimental results? Experiment T5 extends the northern domain boundary by 55 km to 32° North, in order to determine if the hydrology at the Kedua Gap is affected. The land bridge at Kedua opens at 9:30 hours and closes at 13:30 hours, for an elapsed time of 4.0 hours. The passage opening time advances by only 6 minutes as compared with T1. If we use 33 m/s wind for the larger domain (experiment T11), the bridge remains open for 7.4 hours (same as T2). Therefore the smaller (standard) domain does not substantially affect the study area.

The peninsula at Abu Sefeh is surrounded by an eastward-pointing bend in the northern extent of the Ballah Lakes (Figure 3). A land bridge does not form here under a strictly eastern wind (Figure 8). Experiments T6 and T7 use a wind direction blowing from 40° North of East, which is aligned with the primary axis of Ballah Lakes. Wind speeds of 28 m/s and 33 m/s do produce an area of dry lake bed between Abu Sefeh and the opposite (eastern) shore, but there is no water remaining on the left (North) side of the crossing. Therefore the passage from Abu Sefeh, while feasible, cannot technically be considered a “land bridge”. We acknowledge that in a more realistic geography there may be small ponds remaining that our grid resolution cannot resolve. The durations of the dry passages from Abu Sefeh are 2.8 hours for T6, and 8.4 hours for T7. Neither T6 nor T7 generate a dry crossing at Kedua.

Stanley and Warne [11] show in their Figure 5E a Lake of Tanis that is bisected by natural levees of the Tanitic branch of the Nile as it passes from the city of Tanis to the outer mouth on the Mediterranean Sea. Experiment T8 analyzes this configuration. The modeled levees form two unbroken ridges 1 m above sea level, 350 m wide, stretching from the inner Tanitic mouth at (31.0675° N, 31.9610° E) to the outer mouth at (31.1953° N, 32.2033° E). Model configuration T8 produces a delay in the opening time of the Kedua land bridge by 6 minutes as compared with T1, for an elapsed crossing time of 3.8 hours. Therefore the suggested presence of the Tanitic natural levees has very little effect on the formation of a land bridge at the Kedua Gap.

Experiment T9 uses a wind blowing from 30° South of East, in order to test the sensitivity of the Kedua Gap crossing to wind direction. No dry passage forms at Kedua. The Gilbana crossing remains feasible, although no water remains on the right (South) side.

Within the scientific literature, the most uncertain element of our geographic reconstruction is the long coastal sandbar running from Damietta to Pelusium (section 2.2). Without this barrier island, the Lake of Tanis is instead the Gulf of Tineh, a broad shallow shelf extending out into the Mediterranean Sea. Experiments T12 and T13 test this configuration, retaining a depth of 2 m for the shelf until the former location of the sandbar is reached, after which the depth increases as in the standard configuration T1. With wind blowing at 28 m/s in T12, there is no dry crossing at either Kedua or Gilbana. From about 11:00 to 12:00 hours, the Gilbana site retains a stream of water about 200 m wide along the northern shore. Without the coastal sandbar, there is too much water pressure and volume transport from the open Mediterranean Sea to form a dry crossing at Kedua under wind stress of 28 m/s.

Experiment T13 produces a dry crossing at Kedua and Gilbana with wind blowing at 33 m/s. The dry passage at Kedua opens at 8:30 hours and closes at 13:06 hours, for an elapsed crossing time of 4.6 hours. The return surge within the Kedua Gap is from North to South, implying that any debris would be carried South of Kedua toward the eastern shore of the paleolagoon. The dry passage at Gilbana opens at 7:54 hours and closes at 13:06, for an elapsed crossing time of 5.2 hours. Under the stronger East wind of T13, the cape at Pelusium acts as a protective shield for the Kedua Gap, deflecting the Mediterranean inflow and allowing the lagoon there to blow completely dry. T12 and T13 demonstrate that the Kedua Gap crossing has a small but important tolerance to variations in the reconstructed topography.

The standard configuration (T1) of the Kedua Gap is 4 km from the tip of the Sethrum peninsula across to Kedua. Experiment T14 narrows this strait to about 3 km by extending the tip of the peninsula 1 km toward Kedua. T14 produces a dry passage that opens at 9:24 hours and closes at 13:24 hours, for an elapsed crossing time of 4.0 hours. When compared with T1, the shorter Kedua Gap advances the opening time by 12 minutes and increases the crossing time by 6 minutes. This geographical variation has only a minor effect on the crossing.

#### 3.2.6 Model Performance

We executed the ROMS model on the bluefire supercomputer at the National Center for Atmospheric Research, using 1 node with 32 processors. Under this configuration ROMS took 120490 CPU seconds and 4610 wall-clock seconds to run experiment T1 for 0–12 simulated hours, and 278024 CPU seconds and 9953 wall-clock seconds to run T1 for 0–24 simulated hours. The CPU ratio here is 0.43, and the wall-clock ratio is 0.46. Hours 0–12 represent flow driven by wind and gravity, while hours 12–24 represent gravity-driven flow alone. Although it would appear that hours 0–12 are computationally easier, these tests were single runs of the ocean model. Bluefire support staff consider performance variations of less than 10% to be statistically insignificant unless they are observed over a large number of runs. The small deviation from 50% is likely due to differences in the load of the bluefire operating system and its shared disk.

## Discussion

In this paper, we utilize a modern ocean model to investigate an interesting hydrodynamic event involving the phenomenon of wind setdown. Under certain circumstances of topography and wind direction, a body of water may separate, leaving an area of exposed seabed between two points of land. Our study location is across the Kedua Gap (30.9812° N, 32.4553° E) from West to East, an area about 3–4 km long and 5 km wide (Figures 3, 4, and 8).

Two sets of experiments are performed using the ROMS: the first set tests the underwater reef hypothesis of Nof & Paldor [2], [3], and Voltzinger & Androsov [4]. Although Voltzinger & Androsov explored the time-dependent aspects of this problem further than Nof & Paldor did, we find that a realistic reef may differ significantly from their idealized ridge of uniform depth. We used a deeper notch in the reef to demonstrate the difficulty of blowing the Suez reef completely dry. Our model results show that the reef takes more than 12 hours to become fully exposed and passable with realistic bathymetry.

The second set of experiments reports on a new study location. We simulate a wind setdown event at the eastern end of the Lake of Tanis, which extended from Damietta to Pelusium during the Egyptian New Kingdom Period (approximately 1250 BC). The archaeological sites here (Hebua, Tell el-Borg) were above sea level and occupied during this historical period [9], [6]. The ROMS hydrodynamic model demonstrates that a gap opens in the waters where the Pelusiac branch of the Nile flowed into the Lake of Tanis. The resulting land bridge extends about 3–4 km eastward to the archaeological site later known as Tell Kedua. The passage is 5 km wide, and it remains open for 4 hours under 28 m/s wind forcing. The crossing remains open for 7.4 hours under 33 m/s winds, but these stronger winds may render walking too difficult for a mixed group of people. The Kedua Gap and its environs present an interesting hydrodynamic phenomenon for those interested in the history and geography of the eastern Nile delta.

## Supporting Information

File S1 KML file containing the Google Earth overlays used to reconstruct the geography of the eastern Nile delta. The polygons and paths specify where the modern terrain should be modified to reflect the topography of the Late Bronze Age (circa 1250 BC).
(0.03 MB XML)
